# Rationale and design of a randomized trial of the dapagliflozin evaluation on atrial fibrillation patients followed Cox-Maze IV: the DETAIL-CMIV study

**DOI:** 10.1093/europace/euad333

**Published:** 2023-11-06

**Authors:** Zhan Peng, Florian Osmanaj, Yunxiao Yang, Kun Hua, Xiubin Yang

**Affiliations:** Department of Cardiovascular Surgery, Beijing Anzhen Hospital, Capital Medical University, Beijing Institute of Heart, Lung and Vessel Disease, 2# Anzhen Road, Cao Yang District, Beijing 100029, China; Department of Cardiovascular Surgery, Beijing Anzhen Hospital, Capital Medical University, Beijing Institute of Heart, Lung and Vessel Disease, 2# Anzhen Road, Cao Yang District, Beijing 100029, China; Department of Cardiovascular Surgery, Beijing Anzhen Hospital, Capital Medical University, Beijing Institute of Heart, Lung and Vessel Disease, 2# Anzhen Road, Cao Yang District, Beijing 100029, China; Department of Cardiovascular Surgery, Beijing Anzhen Hospital, Capital Medical University, Beijing Institute of Heart, Lung and Vessel Disease, 2# Anzhen Road, Cao Yang District, Beijing 100029, China; Department of Cardiovascular Surgery, Beijing Anzhen Hospital, Capital Medical University, Beijing Institute of Heart, Lung and Vessel Disease, 2# Anzhen Road, Cao Yang District, Beijing 100029, China

**Keywords:** Dapagliflozin, Cox-Maze IV, Atrial fibrillation, Recurrence

## Abstract

**Aims:**

Dapagliflozin has been widely used for the treatment of type 2 diabetes mellitus (T2DM) and heart failure (HF). However, data concerning the association between dapagliflozin and the recurrence of atrial fibrillation (AF), especially in patients following Cox-Maze IV (CMIV), are rare. We aim to explore the effect of dapagliflozin on the recurrence of AF after CMIV with and without T2DM or HF.

**Methods and results:**

The study of dapagliflozin evaluation in AF patients followed by CMIV (DETAIL-CMIV) is a prospective, double-blind, randomized, placebo-controlled trial. A total of 240 AF patients who have received the CMIV procedure will be randomized into the dapagliflozin group (10 mg/day, *n* = 120) and the placebo group (10 mg/day, *n* = 120) and treated for 3 months. The primary endpoint is any documented atrial tachyarrhythmia (AF, atrial flutter or atrial tachycardia) lasting 30 s following a blanking period of 3 months after CMIV.

**Conclusion:**

DETAIL-CMIV will determine whether the sodium-glucose cotransporter-2 inhibitor dapagliflozin, added to guideline-recommended post-operative AF therapies, safely reduces the recurrence rate of AF in patients with and without T2DM or HF.

## Graphical Abstract

**Graphical Abstract:**
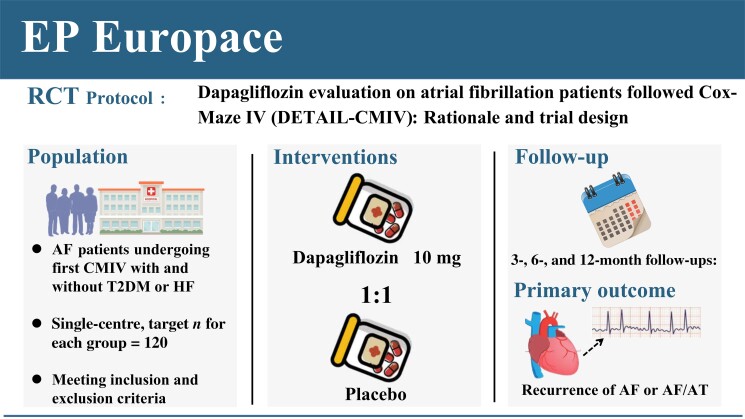

## Introduction

Atrial fibrillation (AF) is the most common arrhythmia. Based on data from the Framingham Heart Study (FHS), the prevalence of AF has increased three-fold over the last 50 years.^1^ The Global Burden of Disease project estimated a worldwide prevalence of AF of approximately 46.3 million individuals in 2016.^2^ Atrial fibrillation is associated with increased mortality that can lead to reduced cardiac output, promote the occurrence of heart failure (HF), and induce abnormal haemodynamic changes in the left atrium, inducing thrombosis.^3^ The Cox-Maze (CM) procedure, which was developed by James Cox in 1987,^4^ is a procedure in which multiple incisions are created in both the left and right atria to eliminate AF while allowing the sinus impulse to reach the atrioventricular node. This procedure became the gold standard for the surgical treatment of AF,^5^ and the latest iteration was termed CMIV, introduced in 2002, which replaced the previous cut-and-sew method by using bipolar radiofrequency ablation and cryoablation to create a biatrial lesion pattern.^6^ However, AF recurrence remains the Achilles heel of the CMIV procedure, with a reported late AF recurrence rate of up to 20–40% after CMIV.^7^

A new oral hypoglycaemic drug, dapagliflozin, a sodium-glucose cotransporter-2 inhibitor (SGLT2i), was confirmed to reduce the risk of cardiovascular adverse events (AEs) and improve coronary heart disease and HF outcomes in multiple clinical trials.^8^ At present, dapagliflozin has been extensively used for type 2 diabetes mellitus (T2DM) and HF. In addition, previous *post hoc* analyses and meta-analyses reported that dapagliflozin can decrease the incidence rate of new-onset AF.^9–11^ Although data on the associations between SGLT2i and the recurrence of AF are relatively limited, recent trials have reported that SGLT2i can achieve greater suppression of AF recurrence after catheter ablation (CA) in T2DM patients.^12,13^ Nonetheless, it remains unknown whether dapagliflozin can improve the recurrence of AF for patients who need the CMIV procedure and whether it can be routinely used after CMIV for AF patients with and without a history of T2DM or HF.

## Objectives

Accordingly, we are conducting a randomized controlled trial to evaluate whether post-operative oral dapagliflozin can improve AF recurrence after CMIV, regardless of concomitant T2DM or HF.

### Study design

**Figure 1:**
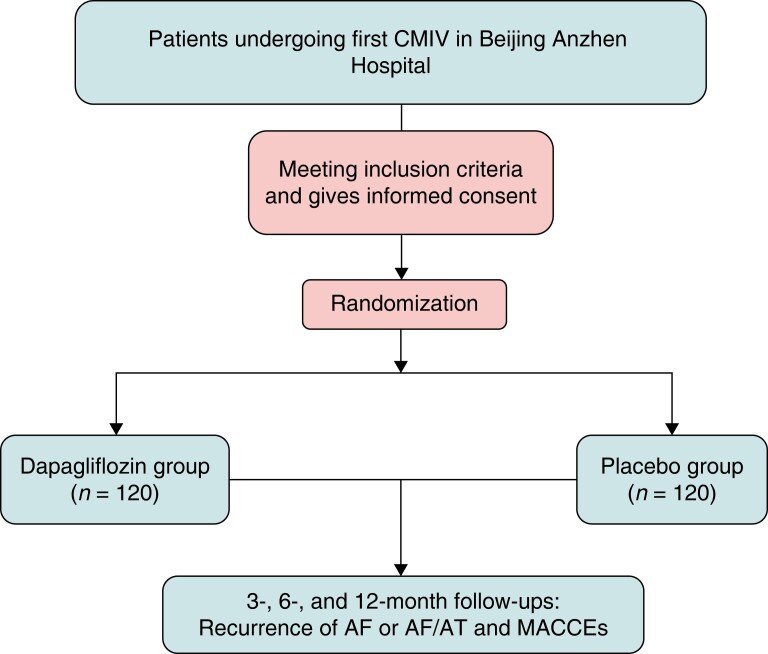
Flowchart of the study. AF, atrial fibrillation; AF/AT, atrial flutter or atrial tachycardia; CMIV, Cox-Maze IV; MACCE, major cardiac or cerebrovascular event.

This is a prospective, double-blind, randomized, placebo-controlled trial. The manufacturer of dapagliflozin did not provide any financial support or take part in the design of the study. This study is investigator initiated, and the study protocol was in accordance with the Declaration of Helsinki and approved by the ethics committee of Beijing Anzhen Hospital (No. KS2023017). The study was registered at http://www.clinicaltrials.gov (NCT05816733). The flowchart of enrolment and follow-up is shown in *Figure 1*.

#### Current status of the trial

The study has not yet begun. The start date of recruitment is expected to be 1 September 2023, and the completion of recruitment is expected by late June 2024.

### Patient population

Patients who were hospitalized in Beijing Anzhen Hospital for CMIV of AF were screened for inclusion with written informed consent was obtained from all participants. Patients aged 18 or older with AF undergoing first-time isolated or concomitant CMIV, regardless of concomitant T2DM or HF, were eligible for inclusion. The CMIV procedure was performed in both concomitant with open heart surgery and a stand-alone procedure at our centre. Patients aged 18 or older with AF undergoing first-time stand-alone or concomitant CMIV, regardless of concomitant T2DM or HF, were eligible for inclusion (concomitant CMIV: symptomatic AF patients undergoing other cardiac surgical procedure; stand-alone CMIV: symptomatic AF patients who have failed medical management and prefer a surgical approach, have recurrent AF after CA or are not candidates for CA). Exclusion criteria were dapagliflozin allergy; hyperthyroidism; acute myocardial infarction, cerebral apoplexy, and other vascular disease during the past 6 months; following heart surgery within the last 3 months; estimated glomerular filtration rate (eGFR) < 45 mL/min; history of oral SGLT2i; estimated survival period < 12 months; pregnant and lactating women; left atrial diameter > 65 mm; and refusing to sign informed consent. Additional inclusion and exclusion criteria are listed in *Table 1*.

**Table 1:** Main inclusion and exclusion criteria of the DETAIL-CMIV trial

**Inclusion criteria**
Age ≥ 18 years Patients who need first-time stand-alone or concomitant Cox-Maze IV procedure (concomitant CMIV: symptomatic AF patients undergoing other cardiac surgical procedure; stand-alone CMIV: symptomatic AF patients who have failed medical management and prefer a surgical approach, have recurrent AF after catheter ablation or are not candidates for catheter ablation) Patients who have the ability and willingness to abide by all the subsequent review and requirements
**Exclusion criteria**
Dapagliflozin allergy Hyperthyroidism Acute myocardial infarction Cerebral apoplexy and other vascular disease during the past 6 months Following heart surgery within the last 3 months eGFR < 45 mL/min History of oral sodium-glucose cotransporter-2 inhibitors Estimated survival period < 12 months Pregnant and lactating women Left atrial diameter > 65 mm Refusing to sign informed consent

### Randomization and blinding

The random allocation sequence was generated with computer-generated random numbers by the study statistician, and sequentially numbered opaque envelopes containing the treatment allocation were utilized to conceal the sequence. Participants will be randomly assigned 1:1 to dapagliflozin (10 mg once a day) or matched placebo with blocked randomization of 10 patients in each block to ensure balance in the treatment groups in case the study needs to be stopped early. Patients and all study personnel (except the independent study statistician) will be kept blinded to the treatment allocation. The trial will adhere to established procedures to maintain separation between the staff that performs the outcome measurements and the staff that delivers the intervention. Staff members who obtain the outcome measurements will not be informed of the treatment group assignment. The intervention staff members who deliver the intervention will not perform the outcome measurements. The dapagliflozin and placebo are packaged in an identical manner with the same labelling, tablet appearance and schedule.

### Surgical procedure

All the CMIV procedures were performed under cardiopulmonary bypass (CPB) with median sternotomy. To reduce the variation among surgeons, we require the CMIV to be performed by qualified surgeons. After the initiation of normothermic CPB, both sets of pulmonary veins are then bluntly dissected, mobilized, and encircled with a urine catheter. Lesions from right atrial (RA) lesion set are performed on the beating heart and with temperature cooled to 34°C. Then, a small purse-string suture is placed at the base of the RA appendage that is wide enough to accommodate one jaw of the bipolar radio frequency (RF) ablation clamp. An ablation lesion is created along the free wall of the right atrium through the purse string down the aortic side of the RA appendage. A vertical right atriotomy is made extending from the intra-atrial septum up towards the atrioventricular groove near the free margin of the heart (at least 2 cm from the free wall ablation). From the inferior aspect of the incision, the RF ablation clamp is used to create ablation lines up to the superior vena cava and down towards the inferior vena cava. A linear cryoprobe is used to create an endocardial ablation line from the superior aspect of this atriotomy down onto the tricuspid annulus at the 2 o’clock position. All cryoablations are performed for 3 min at a temperature below −60°C. The linear cryoprobe is then inserted through the previously placed purse-string suture, and an endocardial ablation line is created down to the tricuspid annulus at the 10 o’clock position. Next, the left atrial lesion set is performed on the arrested heart after aortic cross-clamping. The heart is retracted, and the left atrial appendage (LAA) is exposed and amputated. Through the amputated appendage, the bipolar RF clamp is used to create a connecting lesion into the left inferior pulmonary vein. The LAA is then oversewn in two layers with a running polypropylene suture. The coronary sinus is marked with methylene blue between the right and the left coronary circulations. A standard left atriotomy is then performed, and the bipolar clamp is used to create ‘roof’ and ‘floor’ lesions from the superior and inferior aspects of the atriotomy to the left superior and inferior pulmonary veins, respectively. The RF ablation clamp is also used to create an ablation from the inferior margin of the atriotomy towards the mitral annulus and across the coronary sinus. As the RF bipolar clamp cannot reach the annulus itself, a bell-shaped cryoprobe is used to make an endocardial lesion to the mitral annulus at the end of the mitral isthmus lesion. To complete the left atrial isthmus ablation, an epicardial cryoablation is performed over the coronary sinus in line with the endocardial lesion. The energy source used for each procedure was AtriCure Inc., West Chester, OH, USA. It was repeated three to four times for every ablation to ensure the transmurality. Concomitant cardiac surgery was performed after ablation; for patients with severe coronary artery disease, coronary artery graft bypass was a priority to ensure sufficient cardioplegic solution perfusion.

### Study intervention and background medication

Following CMIV, patients will be randomly assigned to oral dapagliflozin therapy or matched placebo in blocks of ten. Dapagliflozin will be initiated on the first morning after CMIV at a dose of 10 mg once daily until 3 months post-operation. The start date of the observation will be set as the first intervention date. Patients in both groups will undergo 24-h post-operative rhythm monitoring and will be treated with continuous intravenous amiodarone as the standard of care.

All patients will be treated with amiodarone post-operatively, and this trial will test dapagliflozin added to the background medication after CMIV. Routine oral amiodarone at 200 mg twice a day will be administered for 3 months when the patients leave the intensive care unit. Low molecular weight heparin combined with warfarin will be used during the early post-operative period and then treated with warfarin alone after the international normalized ratio (INR) reaches the ideal value (INR of 2.0–3.0).

### Outcome measurement

Primary endpoint is any documented atrial tachyarrhythmia [AF, atrial flutter or atrial tachycardia (AF/AT)] lasting 30 s following a blanking period of 3 months after CMIV.

Secondary endpoints are as follows: (i) any documented atrial tachyarrhythmia [AF, (AF/AT)] at the 6-month follow-up monitoring session after CMIV; (ii) any documented atrial tachyarrhythmia [AF, (AF/AT)] at the 12-month follow-up monitoring after CMIV; and (iii) major adverse cardiac or cerebrovascular events (MACCEs, including cardiovascular death, malignant arrhythmias, and stroke) at 6 or 12 months after CMIV.

### Data collection

Study visits are scheduled to occur before randomization and at 3, 6, and 12 months; a detailed medical history, a routine blood pressure measurement and physical examination, laboratory testing, and 48- to 72-h Holter monitoring will be conducted at each visit. All patients with arrhythmia-related symptoms, especially after the blanking period, will be encouraged to undergo electrocardiography (ECG) during their symptomatic periods, and such events will be registered with the study investigator. In addition, patients who experience longer-lasting arrhythmia symptoms will be instructed to contact local hospitals and undergo cardioversion within 48 h, and additional monitoring (e.g. extra Holter or event monitoring) will be performed for patients with arrhythmia symptoms who cannot be monitored during an acute episode. All ECGs and Holter monitoring will be reviewed by a blinded study investigator. Peri-operative clinical and baseline data, such as the type of AF, medical history, laboratory results, complications, cardiac ultrasound features, general information about the operation, a record of surgical ablation, post-operative complications, and pre-operative medication, will be collected through the electronic medical record database.

### Adverse events monitoring and interim analysis

Adverse events will be constantly monitored by investigators during the process of the study through regular medical check-ups, and the details will be immediately reported to the principal investigator once an AE occurs, regardless of the causal relationship with dapagliflozin. Only serious AEs and AEs of interest or those leading to premature study drug discontinuation, study drug interruption, or dose reduction are recorded. Adverse events of interest include volume depletion, renal events, major hypoglycaemia, and potential diabetic ketoacidosis.

The collection of cardiovascular events and other serious AEs will be performed by two well-trained staff members, and they will follow up specifically for these events by telephone every month for every participant with a phone number who has given prior consent during enrolment. In addition, participants will also be taught and asked to report whether they have experienced any cardiovascular events or other serious AEs. All captive cardiovascular events and other serious AEs will be adjudicated and graded by two well-trained staff members in order to reach a consensus.

Once 75% of the primary events are confirmed, an interim analysis will be performed by using a Haybittle–Peto rule, and if the superiority of dapagliflozin over placebo is demonstrated for the primary outcome with a sided level of 0.001, early termination of the trial can be recommended. The significance level of the final analysis will be determined by the Haybittle–Peto rule, depending on the actual number of events and the timing of the interim analysis.

### Sample size estimation

According to our published work, the recurrence rate of AF after CMIV is 37.9% in patients without oral dapagliflozin therapy.^14^ A recent study showed that dapagliflozin reduced the recurrence rate of AF by 17.1% after RFCA.^13^ We assume a more progressive effect of 20% relative risk reduction. The two-proportion test power analysis was performed by using PASS 15 software (Veraartlaan 12, 2288GM, Rijswijk, the Netherlands), and the algorithms were as follows: (i) power calculation method: normal approximation; (ii) alternative hypothesis: two sided; (iii) test type: *Z*-test (unpooled); (iv) power: 0.9; (v) alpha: 0.05; (vi) group allocation: equal (*N*1 = *N*2); (vii) input type: proportions; (viii) P1 (Group 1 proportion|H1): 0.821; and (ix) P2 (Group 2 proportion): 0.621. Therefore, the estimated sample size required to detect a significant difference in the chi-square test between the two groups under the conditions of a significance level of 5% on both sides and a power of 90% is 101 cases per group. Assuming a dropout rate of 10%, the target number of enrolled patients has been set at 120 cases per group for a total of 240 cases for the two groups.

### Statistical analysis

Analyses will be performed according to the intention-to-treat principle. Patients who are lost or die before completing the 3-month follow-up visit will be excluded from the analysis of the primary endpoint. The proportions of patients with recurrence at follow-up in the dapagliflozin group and the placebo group will be compared using the chi-square test. In general, the last date of contact for each patient will be considered the censoring date for those without any primary outcome event. The cumulative incidence of the first occurrence of any event in the primary endpoint will be plotted by the Kaplan–Meier curve. In addition, the logistic regression model will be used to assess possible predictors for recurrence at follow-up, and pre-specified relevant clinical characteristics will be tested first in a univariate and then in a multivariate-adjusted model.

Continuous variables will be expressed as the mean ± standard deviation or the median and the interquartile range, and Student’s *t*-test, the Mann–Whitney test, and the Wilcoxon rank sum test will be used for comparisons where appropriate. The chi-square test will be performed to analyse categorical variables, which are expressed as frequencies and proportions, and Fisher’s exact test will be used as appropriate. All tests will be two sided, and *P* < 0.05 will be considered to indicate statistical significance. Statistical analyses will be performed by SPSS software (version 26.0).

## Discussion

Dapagliflozin has emerged as a powerful agent to reduce the incidence of cardiovascular events in patients with T2DM and HF. Although the mechanisms of dapagliflozin with cardiovascular events have not been fully demonstrated, studies have shown that dapagliflozin can reduce the production of the inflammatory cytokines interleukin-6 and tumour necrosis factor-α and limit macrophage infiltration in a normoglycaemic rabbit model, which exerts its anti-inflammatory effect.^15^ In addition, Durak *et al*.^16^ showed that dapagliflozin plays an important role in cardioprotective effects through augmentation of mitochondrial function and oxidative stress followed by improvement of fusion–fission proteins. More importantly, dapagliflozin can directly inhibit myocardial NHE-1 and reduce the mRNA expression of NHE-1 in cardiac fibroblasts, which reduces the concentration of Na^+^ and Ca^2+^ in cardiomyocytes to maintain the rhythm of the heart.^17,18^ Herat *et al*.^19^ reported that dapagliflozin can inhibit the abnormal activation of sympathetic nerves by reducing the norepinephrine level and tyrosine hydroxylase staining area in mice. In addition, Sato *et al*.^20^ found that dapagliflozin can reduce epicardial fat, which mediates the release of multiple inflammatory factors resulting in direct heart damage and plays an important role in new-onset AF. If one or more of these mechanisms are operative, then dapagliflozin may also be beneficial in AF patients who need CMIV procedures without T2DM or HF. The DETAIL-CMIV study will test this hypothesis by assessing whether dapagliflozin safely reduces the recurrence of AF in a broad spectrum of patients with and without T2DM or HF.

The relationship between the incidence of new-onset AF and SGLT2i treatment has been previously reported. Early retrospective studies showed that there was no significant difference between SGLT2i treatment and other hypoglycaemic agents,^21,22^ and a meta-analysis also reported that there was no significant difference between the incidence of AF for patients with SGLT2i treatment.^23^ However, this meta-analysis was performed before multicentre, and large clinical trials of SGLT2i were reported. In contrast, a recent *post hoc* analysis from the DECLARE-TIMI 58 trial showed that dapagliflozin reduced the incidence of AF in patients with T2DM.^9^ In addition, Ling *et al*.^24^ found that SGLT2is were related to a lower incidence of new-onset AF than DPP-4 inhibitors. Engström *et al*.^25^ recently performed a cohort study that used nationwide registers in Denmark, Norway, and Sweden and reported that the use of SGLT2 inhibitors (*n* = 79 343), compared with GLP-1 receptor agonists (*n* = 57 613), was associated with a modestly reduced risk of new-onset AF. Sfairopoulos *et al*.^10^ performed a meta-analysis to investigate the effects of SGLT2i on the incidence of AF and/or atrial flutter (AFL) in heart failure with reduced ejection fraction (HFrEF) patients and found that SGLT2i therapy was associated with a significant reduction in the risk of AF and AF/AFL in patients with HFrEF. Li *et al*.^11^ conducted a meta-analysis and found that SGLT2i can decrease the incidence of AF in patients with diabetes mellitus. In addition, Okunrintemi *et al*.^26^ performed a meta-analysis that reported the same conclusion in both diabetic and non-diabetic patients, which included randomized cardiovascular or renal outcome trials. Similarly, this conclusion was also reported by other meta-analyses, which included HF or chronic kidney disease patients.^27,28^ The above studies confirm that SGLT2i can decrease the incidence of AF regardless of whether patients have HF or T2DM. However, data on the associations between SGLT2i and the recurrence of AF are relatively limited. Kishima *et al*.^12^ recently performed a small randomized controlled study comparing the suppressive effect of tofogliflozin vs. dipeptidyl peptidase-4 inhibitors on AF recurrence after CA in T2DM patients and found that tofogliflozin achieved greater suppression of AF recurrence after CA in patients with T2DM, and there was a small retrospective study that included 79 patients treated with dapagliflozin. Luo *et al*.^13^ found that dapagliflozin was associated with a significant reduction in the risk of recurrent atrial arrhythmias after CA in patients with T2DM. However, it remains unknown whether dapagliflozin can improve the recurrence of AF in patients who need the CMIV procedure and whether SGLT2 inhibitors should be part of the standard treatment for AF after CMIV.

In conclusion, the DETAIL-CMIV study is the first dedicated clinical trial to demonstrate the preventive effects of dapagliflozin on the recurrence of AF in patients who need CMIV procedures with and without T2DM or HF. The results of this study may have a large impact on the treatment application of dapagliflozin.

## Data Availability

All relevant data are within the manuscript and its Supporting Information files.

## Clinical trial registration number

NCT05816733.
